# HER2 Phosphorylates and Destabilizes Pro-Apoptotic PUMA, Leading to Antagonized Apoptosis in Cancer Cells

**DOI:** 10.1371/journal.pone.0078836

**Published:** 2013-11-13

**Authors:** Richard L. Carpenter, Woody Han, Ivy Paw, Hui-Wen Lo

**Affiliations:** 1 Division of Surgical Sciences, Department of Surgery, Duke University School of Medicine, Durham, North Carolina, United States of America; 2 Duke Cancer Institute, Duke University School of Medicine, Durham, North Carolina, United States of America; 3 Duke Center for RNA Biology, Duke University School of Medicine, Durham, North Carolina, United States of America; Indiana University School of Medicine, United States of America

## Abstract

HER2 is overexpressed in 15–20% of breast cancers. HER2 overexpression is known to reduce apoptosis but the underlying mechanisms for this association remain unclear. To elucidate the mechanisms for HER2-mediated survival, we investigated the relationship between HER2 and p53 upregulated modulator of apoptosis (PUMA), a potent apoptosis inducer. Our results showed that HER2 interacts with PUMA, which was independent of HER2 activation. In addition, we observed that HER2 interacted with PUMA in both mitochondrial and non-mitochondrial compartments. We next examined whether HER2 phosphorylates PUMA. Notably, PUMA tyrosine phosphorylation has never been reported. Using an intracellular assay, we found PUMA to be phosphorylated in breast cancer cells with activated HER2. Via cell-free HER2 kinase assay, we observed that PUMA was directly phosphorylated by HER2. Activation of HER2 decreased PUMA protein half-life. To identify which of the three tyrosines within PUMA are targeted by HER2, we generated three PUMA non-phosphorylation mutants each with a single Tyr→Phe substitution. Results indicated that each PUMA single mutant had lost some, but not all phosphorylation by HER2 indicating that HER2 targets all three tyrosines. Consequently, we created an additional PUMA mutant with all three tyrosines mutated (TM-PUMA) that could not be phosphorylated by HER2. Importantly, TM-PUMA was found to have a longer half-life than PUMA. An inverse association was observed between HER2 and PUMA in 93 invasive breast carcinoma samples. We further found that TM-PUMA suppressed growth of breast cancer cells to a greater degree than PUMA. Also, TM-PUMA had a stronger propensity to induce apoptosis than PUMA. Together, our results demonstrate, for the first time, that PUMA can be tyrosine phosphorylated and that HER2-mediated phosphorylation destabilizes PUMA protein. The HER2-PUMA interplay represents a novel mechanism by which PUMA is regulated and a new molecular basis for HER2-mediated growth and survival of cancer cells.

## Introduction

Breast cancer rates are declining but it remains a significant public health threat. Current estimates indicate there will be 300,000 new cases and 40,000 deaths from breast cancer in 2013 [1]. It is estimated that there will be more new breast cancer cases in women than any other type of cancer in 2013 [1]. HER2 is overexpressed in 15–20% of human breast cancers and this overexpression is associated with poor patient outcomes including decreased overall survival, increased tumor relapse, and more aggressive disease [2]–[4]. HER2 activation occurs by heterodimerization with other ERBB family receptors, such as heregulin binding to HER3 that will then heterodimerize with HER2 to activate downstream HER2 pathways [5].

Overexpression of HER2 is known to reduce apoptosis. Pro-apoptotic and anti-apoptotic Bcl-2 proteins control the intrinsic apoptotic pathway at the mitochondria. Positive correlations have been found between HER2 expression and anti-apoptotic proteins such as Bcl-xL, Mcl-1, and Bcl-2 [6]–[8]. In addition, forced expression of HER2 caused increased protein levels of anti-apoptotic proteins such as Bcl-2 and Bcl-xL while inhibition of HER2 reduced Mcl-1 and increased Bax expression [6], [7], [9]. HER2 can also activate PI3K-AKT and ERK1/2 signaling, which can regulate apoptosis by controlling gene expression, such as upregulation of survivin, and post-translational regulation, such as phosphorylation and inactivation of pro-apoptotic Bad [10], [11]. HER2 regulation of apoptosis has primarily been observed to be mediated by downstream signaling while direct regulation of Bcl-2 proteins by HER2 has not been assessed.

The *PUMA* gene was first identified in 2001 [12], [13] as a screen for transcriptional targets of p53. The *BBC3* gene was identified soon after by yeast two-hybrid screening and cDNA for this gene matched that of PUMA [14]. This later discovery of the *BBC3* gene also established that PUMA expression could be induced by apoptotic stimuli independent of p53 [14]. PUMA contains two functional domains on the C-terminus, the BH3 domain and the mitochondrial localization signal (MLS) [15], [16]. Functional activity of PUMA is initiated by protein targeting to the outer mitochondrial membrane where PUMA interacts with anti-apoptotic Bcl-2 family members inhibiting their suppression of Bax and Bak [12], [17]. Inhibition of anti-apoptotic Bcl-2 family members leads to activation of pro-apoptotic proteins Bax/Bak triggering mitochondrial outer membrane permeabilization (MOMP) and release of cytochrome C [12], [16], [17]. Cytoplasmic cytochrome C ultimately forms the apoptosome leading to activation of effector caspases 3/9 and apoptosis. Loss of PUMA activity has been associated with multiple cancer types. Deletion of a portion of chromosome 19, where the *PUMA* gene is located, has been reported in multiple cancer types [13], [18], [19]. In addition, *PUMA* is a p53-inducible gene and p53 has mutations in more than 40% of cancers [20]. Consequently, impaired PUMA induction has been observed with p53 mutation or deletion [13], [21]. Also, cancer cells with PUMA deleted have high resistance to p53-inducible therapies such as DNA-damaging agents, UV, and gamma-irradiation among others [19]. However, a number of studies have reported that PUMA expression can be induced by p53-independent mechanisms [19], [22]–[25].

A direct link between HER2 and PUMA has not been investigated. Also unknown is whether PUMA undergoes tyrosine phosphorylation. In this study, we discovered a novel finding that PUMA can be phosphorylated on tyrosine residues directly by HER2. Furthermore, PUMA phosphorylation by HER2 leads to PUMA destabilization and cell survival. We also show that a PUMA mutant that cannot be phosphorylated on tyrosine residues (TM-PUMA) has an enhanced ability to induce apoptosis. Taken together, our study uncovered a novel HER2→PUMA signaling axis that represents a novel mechanism by which PUMA protein and PUMA-mediated cell death are regulated. Our findings also provide evidence implicating PUMA down regulation as a new molecular basis for HER2-mediated growth and survival.

## Materials and Methods

### Cells and Cell Culture

MDA-MB-453, MCF-7, BT-474, and SK-BR3 human breast cancer cell lines were obtained from American Type Culture Collection, ATCC (Manassas, VA). MDA-MB-453 cells were maintained in Leibovitz L-15 medium supplemented with 10% fetal bovine serum (FBS) at 0% carbon dioxide. MCF-7 cells were maintained in MEM medium supplemented with 10% FBS, 1 mM sodium pyruvate, 0.1 mM non-essential amino acids, and 10 µg/mL bovine insulin. BT-474 cells were maintained in DMEM medium supplemented with 10% FBS. SK-BR3 cells were maintained in McCoy’s 5a medium supplemented with 10% FBS. MCF-7/HER2 cells were maintained in MEM medium supplemented with 10% FBS, 1 mM sodium pyruvate, 0.1 mM non-essential amino acids, 10 ug/mL bovine insulin, and 350 µg/mL G418.

### Chemicals and Reagents

All chemicals were purchased from Sigma (St. Louis, MO) unless otherwise stated. Cycloheximide was purchased from Amresco (Solon, OH), MG132 was purchased from CalBiochem (San Diego, CA), anisomycin was purchased from Enzo Life Sciences (Farmingdale, NY), and Lapatinib was purchased from LC Laboratories (Woburn, MA). Tubulin, β-actin, and IgG antibodies were purchased from Sigma. HA antibody was purchased from Roche (Indianapolis, IN), PARP-1 antibody was purchased from Santa Cruz Biotechnology (Santa Cruz, CA), COX IV antibody was purchased from Abcam (Cambridge, MA), and 4G10 antibody was purchased from Millipore (Billerica, MA). PUMA, HER2, and phosphor-HER2 (Y1248) antibodies were purchased from Cell Signaling Technologies (Danvers, MA).

### Plasmids

The pHA-PUMA plasmid was kindly provided by Dr. Bert Vogelstein via Addgene plasmid 16588 [13]. Generation of single mutant PUMAs (Y58F-PUMA, Y152F-PUMA, and Y172F-PUMA) as well as the triple mutant (Y58F/Y152F/Y172F-PUMA) was done using a QuikChange Site-Directed Mutagenesis kit (Agilent Technologies, Santa Clara, CA) per manufacturer’s instructions. Primers used for mutagenesis were the following: Y58F-Forward 5′-TGCCCGCTGCCTTTCTCTGCGCCCCCA-3′, Y58F-Reverse 5′-TGGGGGCGCAGA GAAAGGCAGCGGGCA-3′, Y152F-Forward 5′-CTCAACGCACAGTTTGAGCGGCGGAGA-3′, Y152F-Reverse 5′-TCTCCGCCGCTCAAACTGTGCGTTGAG-3′, Y172F-Forward 5′-TCACCTGGAGGGTCCTGTTCAATCTCATCAT-3′, and Y172F-Reverse 5′-ATGATGAGATTGAACAGGACCCTCCAGGGTGA-3′. Mutation was confirmed by sequencing.

### Immunoprecipitation/Western Blotting (IP/WB)

Cells were lysed with RIPA buffer (50 mM Tris, 150 mM NaCl, 1 mM EDTA, 1% NP-40, 0.1% SDS, 1% sodium deoxycholate) supplemented with protease/phosphatase inhibitors followed by sonication and collection of supernatant. Whole cell extracts were pre-cleared with 1 µg rabbit IgG and 20 µL protein-A-agarose for 1 hr at 4°C. Cleared lysates were then incubated with 1 µg HER2 or PUMA antibody or control rabbit IgG at 4°C overnight with agitation. Protein A-agarose was then added and incubated at 4°C for 60 minutes with agitation. Protein A-agarose pellets were collected and washed multiple times with RIPA buffer at 4°C. Washed pellets were boiled and subjected to SDS-PAGE and immunoblotting as described previously [26]. Determination of PUMA tyrosine phosphorylation was determined by immunoprecipitation of PUMA followed by immunoblotting using anti-phospho-tyrosine 4G10 Platinum antibody (Millipore).

### Cell-free HER2 Kinase Assays

Recombinant human PUMA protein (Origene, Rockville, MD) was dephosphorylated with recombinant human PTP1B protein at 30°C followed by PTP1B inactivation at 65°C. Dephosphorylated PUMA was then incubated with recombinant human HER2 protein (Promega, Madison, WI) and ATP at 30°C. Sample was then boiled and subjected to SDS-PAGE and immunoblotting using anti-phospho-tyrosine 4G10 Platinum antibody (Millipore).

### Immunoprecipitation-kinase Assay

Cells were transfected with HA-tagged PUMA plasmids and cell lysates were collected as described above. Immunoprecipitated was done with HA antibody as described above. Following washes, protein A-agarose pellets were dephosphorylated with recombinant human PTP1B followed by incubation with recombinant human HER2 as described above. Samples were then boiled and subjected to SDS-PAGE and immunoblotting using anti-phospho-tyrosine 4G10 Platinum antibody (Millipore).

### Colony Formation Assays

Following transfection of indicated plasmids cells were seeded into 6-well culture plates to determine anchorage-dependent clonogenic growth as we described previously [27]. Following transfection, cells were also seeded into 6-well plates with agarose to determine anchorage-independent clonogenic growth. Wells pre-coated with a bottom layer of 0.5% agarose and cells were seeded into top layer with 0.35% agarose. After 2–4 weeks, colonies with or without agarose were stained with crystal violet blue solution (Sigma) for 1 hr and colonies were counted under a microscope. Experiments were performed in triplicate.

### Assessment of Apoptosis

Cells were transfected with indicated plasmids, treated with indicated compounds, and harvested with trypsin/EDTA. Apoptosis was then determined using FITC-Annexin V/propidium iodide detection kit from BD Pharmingen (San Jose, CA) per manufacturer’s instructions. Cells were then analyzed by flow cytometry using a BD FACSCalibur flow cytometer. PARP-1 cleavage was determined using immunoblotting of cell lysates following indicated transfection and treatment.

### Determination of PUMA Mitochondrial Levels

Mitochondrial fractionation was performed using an assay kit from Pierce/Thermo Scientific (Rockford, IL), according to manufacturer’s instructions as we have described previously [26]. Briefly, protein was isolated from the mitochondrial extract (ME) and the non-mitochondrial extract (NME). The ME and NME were then subjected to SDS-PAGE and immunoblotting. Band intensities were then measured using NIH ImageJ software. The extent of PUMA mitochondrial localization (mtPUMA Index) was computed using band densities with the following equation:
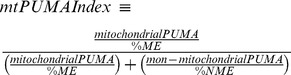
%ME is the percent of the total ME loaded and %NME is the percent of the total NME loaded for immunoblotting.

### Immunohistochemistry of Clinical Tumor Samples

Slides were purchased from US Biomax (Rockville, MD). Assessment of HER2 intensity (0–3+) was completed by US Biomax. PUMA detection was conducted as we described previously [28]. Slides were incubated with PUMA antibody (Cell Signaling Technology, Danvers, MA). Histologic scores (H-Scores) were computed from both percent positivity (A%, A = 1–100) and intensity (B = 0–3+) using the following equation: H-Score = A×B. Chi-square analysis was used to determine relationship between HER2 intensity and PUMA H-Score.

### Statistical Analyses

Data are presented as Mean ± SE. Differences were determined via One-Way ANOVA with Tukey’s post-hoc test or student’s t-test where appropriate. Chi-Square analysis was performed for the IHC results. Significance was set at p<0.05.

## Results

### HER2 Physically Associates with PUMA

**Figure 1:**
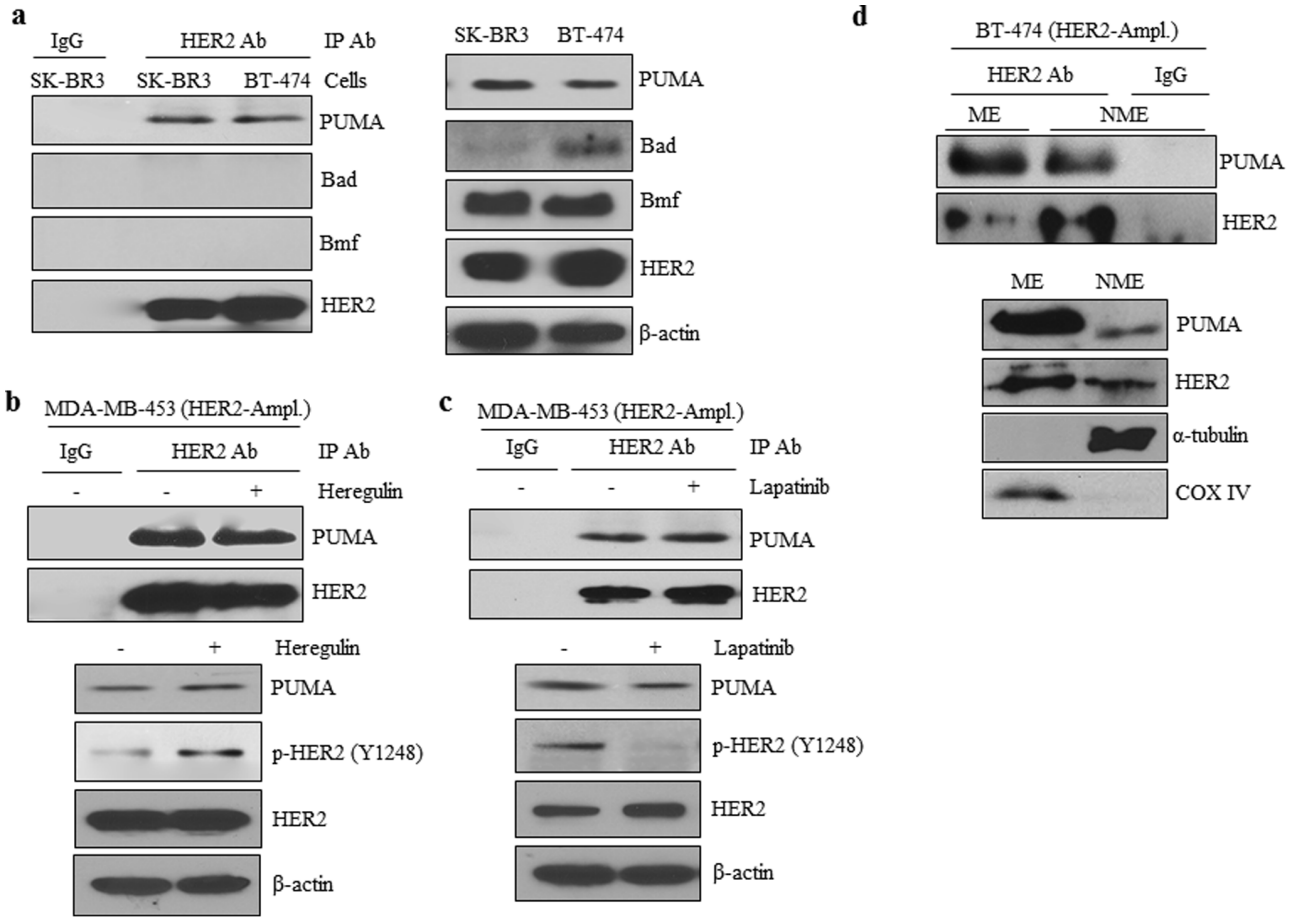
HER2 Directly Interacts with PUMA. a) SKBR3 and BT-474 cells were lysed and total protein subjected to immunoprecipitation with either control IgG or HER2 antibodies followed by immunoblotting with indicated antibodies. Whole cell lysates were also subjected to immunoblotting with indicated antibodies. b) MDA-MB-453 cells were incubated in serum-free medium for 16 hrs followed by treatment with heregulin (100 ng/mL) for 30 minutes. Cells were then lysed and total protein was subjected to immunoprecipitation with either control IgG or HER2 antibodies followed by immunoblotting with indicated antibodies. Whole cell lysates were also subjected to immunoblotting with indicated antibodies. c) MDA-MB-453 cells were incubated with lapatinib (10 µM) for two hrs. Cells were then lysed and total protein was subjected to immunoprecipitation with either control IgG or HER2 antibodies followed by immunoblotting with indicated antibodies. Whole cell lysates were also subjected to immunoblotting with indicated antibodies. d) The mitochondrial (ME) and non-mitochondrial extract (NME) were isolated from BT-474 cells and both extracts were subjected to immunoprecipitation with indicated antibodies. ME and NME were also subjected to immunoblotting with indicated antibodies.

To investigate whether HER2 has any interplay with PUMA, we first assessed whether HER2 can physically interact with PUMA using immunoprecipitation/western blotting (IP/WB). We used SK-BR3 and BT-474 breast cancer cells as they overexpress HER2 due to HER2 gene amplification. We immunoprecipitated HER2 from these cells and found that PUMA could be detected with HER2 pull down in both cell lines (Figure 1a). This indicated a novel finding that HER2 physically interacts with PUMA. It is worth noting that we did not detect an interaction between HER2 and Bad or Bmf, other BH3-only proteins, suggesting the interaction with HER2 is specific to PUMA (Figure 1a). We next assessed whether HER2 kinase activity was required for the interaction with PUMA. For this, cells were treated with or without heregulin as a means of activating HER2 kinase activity. Of note, HER2 does not have obvious ligands and relies on binding to heregulin-bound HER3 for activation; HER3 does not have kinase activity [5]. As shown in Figure 1b, HER2 was activated by heregulin but this did not significantly change the interaction of HER2 with PUMA. Cells were then treated with or without lapatinib, which inhibits HER2 activation [29]. Lapatinib decreased HER2 activation but also did not significantly affect the interaction of HER2 and PUMA (Figure 1c). Collectively, these data indicate that HER2 can physically interact with PUMA and this interaction is not dependent on kinase activity of HER2.

PUMA primarily localizes to the mitochondria as it contains a mitochondrial localization signal [12], [16] but PUMA has also been observed to promote apoptosis without mitochondrial localization [15]. In addition, HER2 is primarily localized to the plasma membrane but has recently been found to localize to the mitochondria where it influences cellular metabolism and promotes resistance of trastuzumab [30]. Therefore, we next determined where PUMA and HER2 physically interact. Mitochondrial (ME) and non-mitochondrial extracts (NME) were isolated from BT-474 cells and immunoblotting for α-tubulin and COX IV confirmed there was effective isolation of mitochondrial and non-mitochondrial fractions (Figure 1d). Figure 1d shows that PUMA and HER2 were detected in both the ME and the NME confirming previous observations [30]. Despite loading of equal amounts of protein (60 µg), there appeared to be greater PUMA and HER2 levels in the ME than the NME. However, this apparent imbalance is due to the fact that 60 µg is 80% of the ME harvested but only 2% of the NME harvested. To determine interaction between HER2 and PUMA, HER2 was immunoprecipitated from equal amounts of the ME and NME followed by immunoblotting. We observed interaction between HER2 and PUMA in both the ME and the NME (Figure 1d). These are the first data indicating HER2 physically interacts with PUMA and that this interaction occurs in and out of the mitochondrial compartment.

### HER2 Directly Phosphorylates PUMA

**Figure 2:**
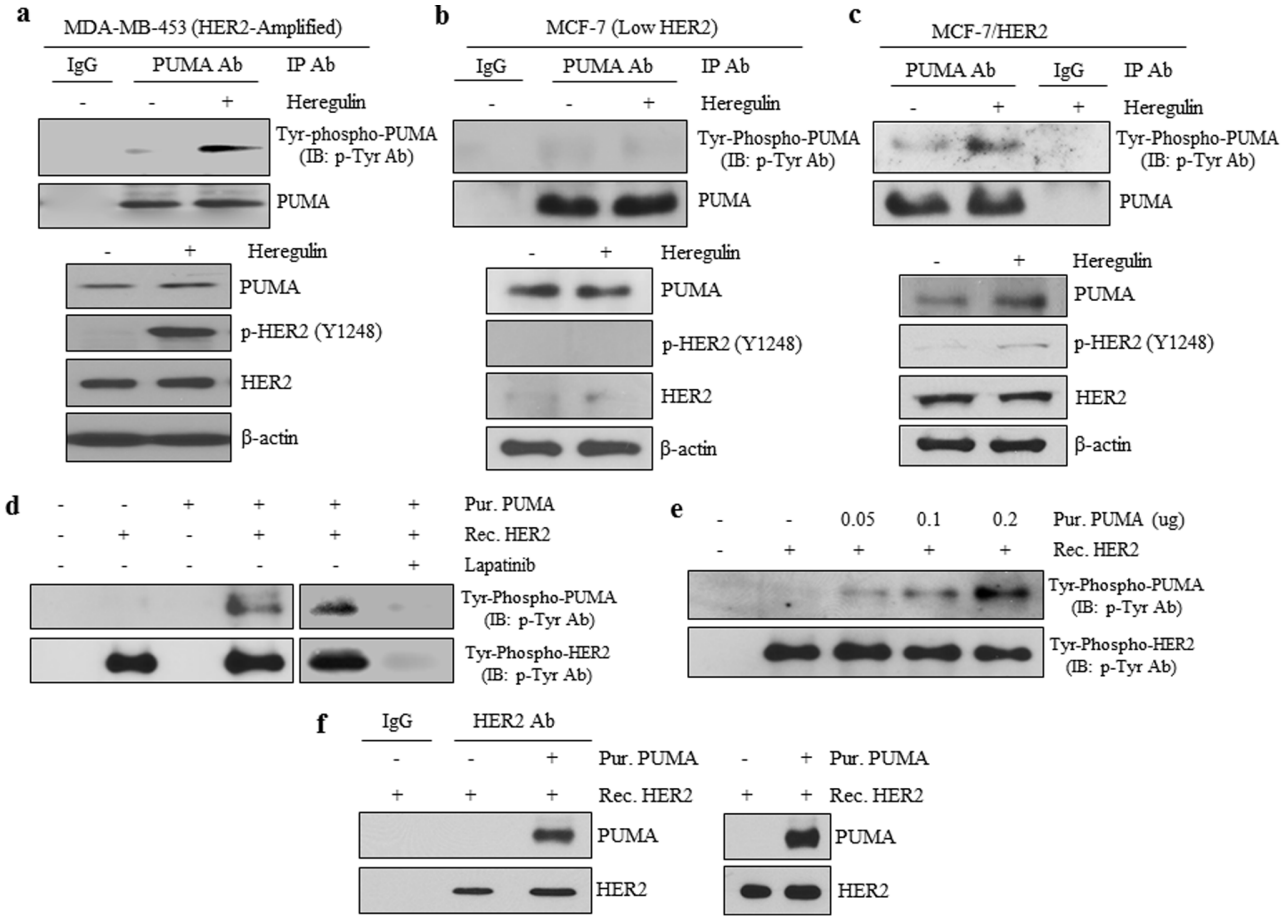
HER2 Directly Phosphorylates PUMA. MDA-MB-453 (a), or MCF-7 (b), or MCF-7/HER2 (c) cells were incubated in serum-free medium for 16 hrs followed by treatment with heregulin (100 ng/mL) for 30 min. Cells were then lysed and total protein was subjected to immunoprecipitation with either control IgG or PUMA antibodies followed by immunoblotting with indicated antibodies. Whole cell lysates were also subjected to immunoblotting with indicated antibodies. Tyrosine-phosphorylated PUMA was detected with 4G10 phospho-tyrosine antibodies. Recombinant PUMA protein was subjected to the HER2 kinase assay (as indicated in the Methods & Materials) in the presence or absence of lapatinib (d) or with increasing levels of recombinant PUMA protein (e). f) Recombinant HER2 was immunoprecipitated in the presence or absence of purified PUMA followed by immunoblotting with indicated antibodies.

Following detection of a direct interaction between HER2 and PUMA, we next determined whether PUMA could be phosphorylated by HER2. To the best of our knowledge, PUMA tyrosine phosphorylation has not been previously reported. To first assess whether PUMA can be tyrosine phosphorylated intracellularly, we starved HER2-overexpressing cells for 16 hrs and then treated the cells with or without heregulin to activate HER2. We subjected the cell lysates to IP/WB using a PUMA antibody for IP and immunoblotted with anti-phospho-tyrosine antibodies. As shown in Figure 2a, tyrosine-phosphorylated PUMA was readily detected in heregulin-stimulated cells which expressed activated phosphorylated HER2 (p-HER2). However, MCF-7 cells, which express low levels of HER2, did not respond to heregulin and did not show significant PUMA tyrosine phosphorylation (Figure 2b). In contrast, MCF-7 cells with stable, forced HER2 overexpression (MCF-7/HER2 cells) shows PUMA tyrosine phosphorylation in response to heregulin (Figure 2c). These results are the first to show that PUMA can be phosphorylated on tyrosine residues and this occurred with HER2 stimulation by heregulin.

We next wanted to determine whether HER2 could directly phosphorylate PUMA. To this end, we used commercially available purified recombinant PUMA and HER2 proteins to perform a cell-free kinase assay. As shown in Figure 2d, PUMA was strongly phosphorylated at tyrosine residues in the presence of HER2. As expected, HER2 underwent auto-phosphorylation. In the presence of lapatinib, HER2 phosphorylation was lost, and consequently, there was no tyrosine phosphorylation of PUMA. We also observed a dose-response increase in tyrosine phosphorylation of PUMA with increasing levels of PUMA protein in the presence of HER2 (Figure 2e). Using IP-WB, we further show that pulldown of recombinant HER2 also results in pulldown of purified PUMA (Figure 2f) confirming HER2 directly associates with PUMA in the context of the cell-free kinase assay. These results show for the first time that PUMA can be phosphorylated at tyrosine residues directly by HER2.

### HER2 Phosphorylates PUMA at Three Tyrosine Residues

**Figure 3:**
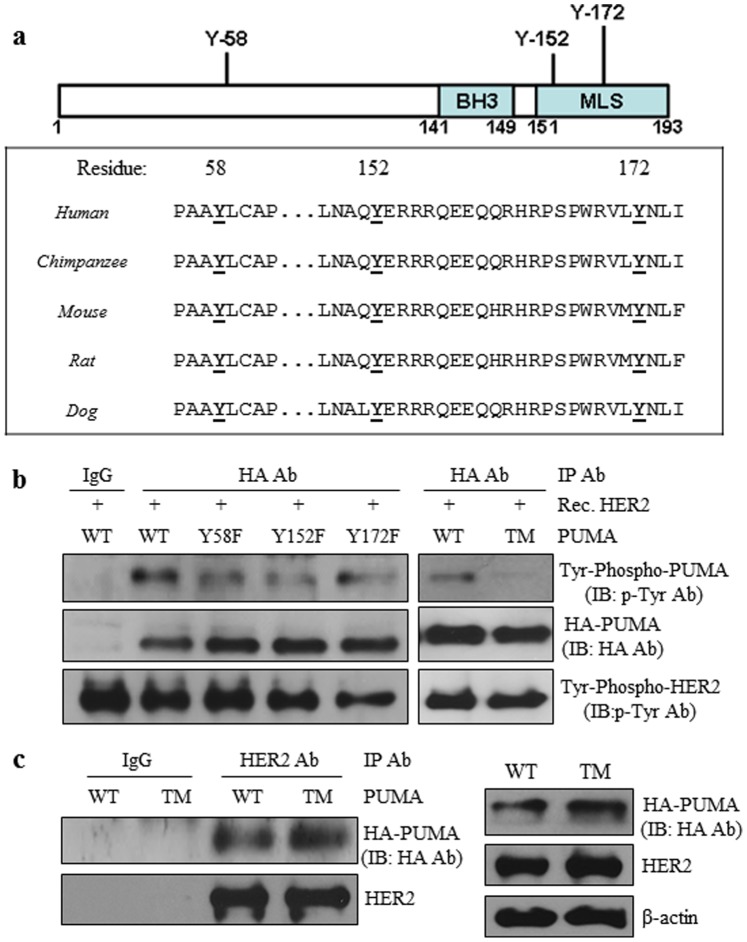
HER2 Phosphorylates Three Tyrosine Residues on PUMA. a) Linear representation of the PUMA protein with each tyrosine, the BH3 domain, and mitochondrial localization signal (MLS) domain indicated (upper panel). Tyrosines 58, 152, and 172 in the PUMA protein is conserved across multiple mammalian species, which are indicated (lower panel). b) Wild-type HA-tagged PUMA protein was mutated so that each tyrosine was changed to phenylalanine (Y58F, Y152F, Y172F) or all tyrosines were mutated (triple mutant: TM). MCF-7 cells were transfected with WT-PUMA or each PUMA mutant and whole cell lysate was subjected immunoprecipitation with either control IgG or HA-directed antibodies. Following immunoprecipitation, the product was subjected to the HER2 kinase assay as indicated in the Materials and Methods section. c) WT-PUMA or TM-PUMA were transfected into MDA-MB-453 cells. Cells were lysed and total protein subjected to immunoprecipitation and immunoblotting with indicated antibodies. Whole cell lysates were also subjected to immunoblotting with indicated antibodies.

A search of the human PUMA protein sequence revealed the presence of three tyrosine residues, namely Y58, Y152, and Y172 (Figure 3a). All three tyrosine residues in PUMA were found to be conserved across multiple mammalian species (Figure 3a), indicating these residues are potentially functionally important. To determine which specific PUMA tyrosine residue(s) that HER2 phosphorylates, we conducted site-directed mutagenesis to mutate each tyrosine (Tyr; Y) to phenylalanine (Phe; F) using an expression vector carrying HA-tagged PUMA as the template. Phenylalanine has the same R group as tyrosine without the oxygen to bind phosphate and, thus, cannot be phosphorylated. These PUMA mutants (Y58F-, Y152F-, Y172F-PUMA), along with wild-type PUMA (WT-PUMA), were expressed in cells, immunoprecipitated using an HA-tag antibody, and subjected to the HER2 kinase assay. As shown in Figure 3B, WT-PUMA was strongly phosphorylated by recombinant HER2 while all of the mutants showed a low level of phosphorylation, indicating that all three tyrosines can be phosphorylated. To fully understand the biological consequences of PUMA tyrosine phosphorylation we created an additional PUMA mutant, a triple mutant PUMA (TM-PUMA), in which all three tyrosines (Y58, Y152, and Y172) were mutated to phenylalanine. Using the cell-free HER2 kinase assay (Figure 3b), WT-PUMA showed phospho-tyrosine bands whereas none were detected with TM-PUMA, indicating the TM-PUMA is not phosphorylated by HER2. To rule out the possibility that TM-PUMA cannot be tyrosine-phosphorylated due to its inability to interact with HER2, we next determined whether TM-PUMA can physically interact with HER2. IP/WB with a HER2 antibody (Figure 3c) demonstrated that HER2 interacted with both WT-PUMA and TM-PUMA equally indicating the lack of TM-PUMA phosphorylation by HER2 is not due to decreased interaction between the two proteins. Taken together, the results in Figures 2 and 3 are the first evidence showing that PUMA undergoes tyrosine phosphorylation and that HER2 can directly phosphorylate PUMA.

### TM-PUMA has a Longer Half-life than WT-PUMA

**Figure 4:**
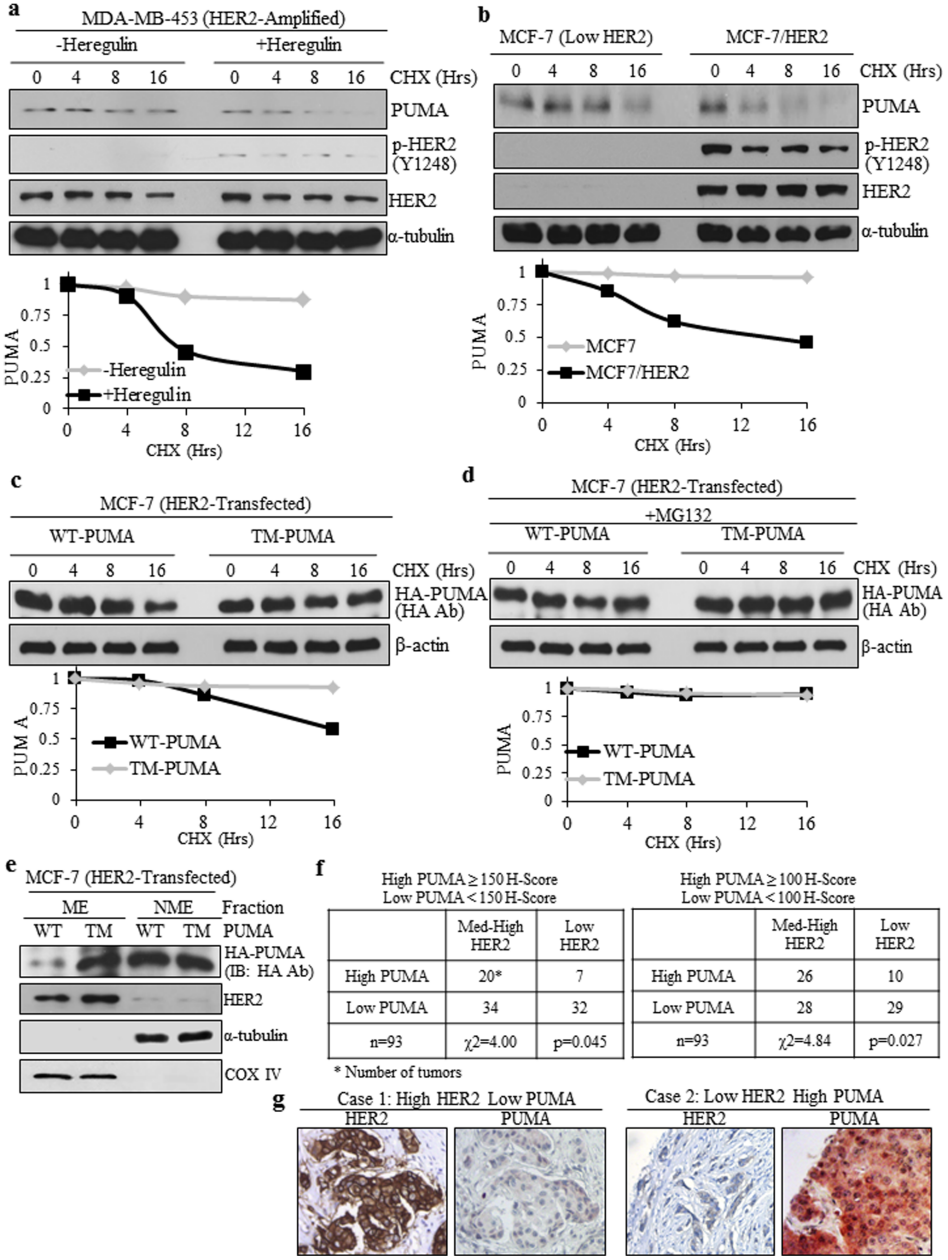
HER2 Phosphorylation Regulates Half-Life and Mitochondrial Levels. a) MDA-MB-453 cells were incubated in serum-free medium for 16 hrs followed by treatment with heregulin (100 ng/mL) and cycloheximide (10 ug/mL). Whole cell lysate was subjected to immunoblotting with indicated antibodies. b) MCF-7 and MCF-7/HER2 cells were incubated in serum-free medium for 16 hrs followed by treatment with heregulin (100 ng/mL) and cycloheximide (10 ug/mL). Whole cell lysate was subjected to immunoblotting with indicated antibodies. c,d) MCF-7 cells were transfected with HER2 and either WT-PUMA or TM-PUMA. Cells were incubated in serum-free medium for 16 hrs followed by treatment with heregulin (100 ng/mL) and cycloheximide (10 ug/mL) without (c) or with MG132 (10 µM) co-treatment (d). Whole cell lysate was subjected to immunoblotting with indicated antibodies. e) MCF-7 cells were transfected with WT-PUMA or TM-PUMA and mitochondria were isolated. ME and NME were subjected to immunoblotting with indicated antibodies. f) Chi-square tables for analysis of clinical cancer samples. Med-High HER2 = 2–3+. Low HER2 = 0–1+. g) Sample IHC images of clinical cancer samples for High HER2+ Low PUMA (case 1) and Low HER2+ High PUMA (case 2).

We next wanted to determine whether PUMA phosphorylation by HER2 altered PUMA stability. To this end, we assessed protein half-life using cycloheximide, which inhibits protein synthesis allowing detection of when proteins are degraded. Cycloheximide is a common method to determine protein stability as several relevant papers have used this method in recent years [31]–[34]. Thus, HER2-overexpressing MDA-MB-453 cells were treated with cycloheximide for up to 16 hrs in the presence or absence of heregulin to activate HER2. As shown in Figure 4a, heregulin induced activation of HER2 in these cells and also led to enhanced PUMA protein degradation. To further examine the stability of PUMA, we assessed PUMA half-life using MCF-7 cells, which have low HER2 expression, or MCF-7/HER2 cells, which have stable overexpression of HER2. Figure 4b shows that PUMA is degraded faster in MCF-7/HER2 cells compared to MCF-7 cells indicating HER2 overexpression reduces PUMA stability. We next determined whether the half-life of TM-PUMA, which cannot be tyrosine phosphorylated, differs from that of WT-PUMA. Cells were transfected with either WT-PUMA or TM-PUMA followed by cycloheximide treatment. As shown in Figure 4c, WT-PUMA levels significantly decreased at 16 hrs whereas TM-PUMA levels did not substantially decline. Following quantification of PUMA band signals and plotting them over time, we found that the half-life for WT-PUMA was approximately 7 hrs whereas that of TM-PUMA was longer than 16 hrs. It has been previously shown that PUMA can be targeted to the proteasome for degradation [31]. To determine if WT-PUMA or TM-PUMA is regulated by the proteasome, we performed the half-life experiment in the presence of the proteasome inhibitor MG132. As shown in Figure 4d, we observed that WT-PUMA half-life could be extended with inhibition of the proteasome confirming previous results [31]. These results suggest HER2-mediated phosphorylation reduces the half-life of PUMA.

We next asked whether TM-PUMA retains the ability to undergo translocalization to the mitochondria where PUMA promotes apoptosis. Thus, WT-PUMA or TM-PUMA were transfected into cells followed by isolation of the ME and NME with subsequent immunoblotting. As Figure 4e indicates, TM-PUMA retained the ability to undergo mitochondrial localization. Furthermore, we observed greater levels of TM-PUMA compared to WT-PUMA in the ME, which was confirmed by calculation of the mtPUMA Index (see Materials and Methods) resulting in 3.3 times more TM-PUMA in the mitochondria than WT-PUMA. A greater TM-PUMA level in the mitochondria is likely the result of enhanced protein stability of TM-PUMA protein in the presence of HER2. Together, these data show that PUMA protein stability is decreased with HER2 activation and blocking PUMA tyrosine phosphorylation enhances PUMA stability and results in greater mitochondrial levels of PUMA.

To assess whether this relationship is maintained *in vivo*, we performed immunohistochemistry on a set of clinical cancer samples (n = 93) to detect HER2 and PUMA. After scoring, we divided the samples into low HER2 (0–1+ intensity) or medium to high HER2 (2–3+ intensity). PUMA was divided into high PUMA (either ≥150 H-Score or ≥100 H-Score) or low PUMA (either <150 H-Score or <100 H-Score). We then performed a chi-square analysis to determine the relationship between HER2 and PUMA expression (Figure 4f). The chi-square analysis using either PUMA barrier (150 H-Score or 100 H-Score) resulted in statistical significance (p = 0.045 and p = 0.027, respectively). These data suggest the tissues with high HER2 expression tend to have lower PUMA expression *in vivo* (Figure 4g) supporting our data from cell lines that HER2 can downregulate PUMA expression.

### TM-PUMA has a Stronger Effect than WT-PUMA on Suppressing Clonogenic Growth

**Figure 5:**
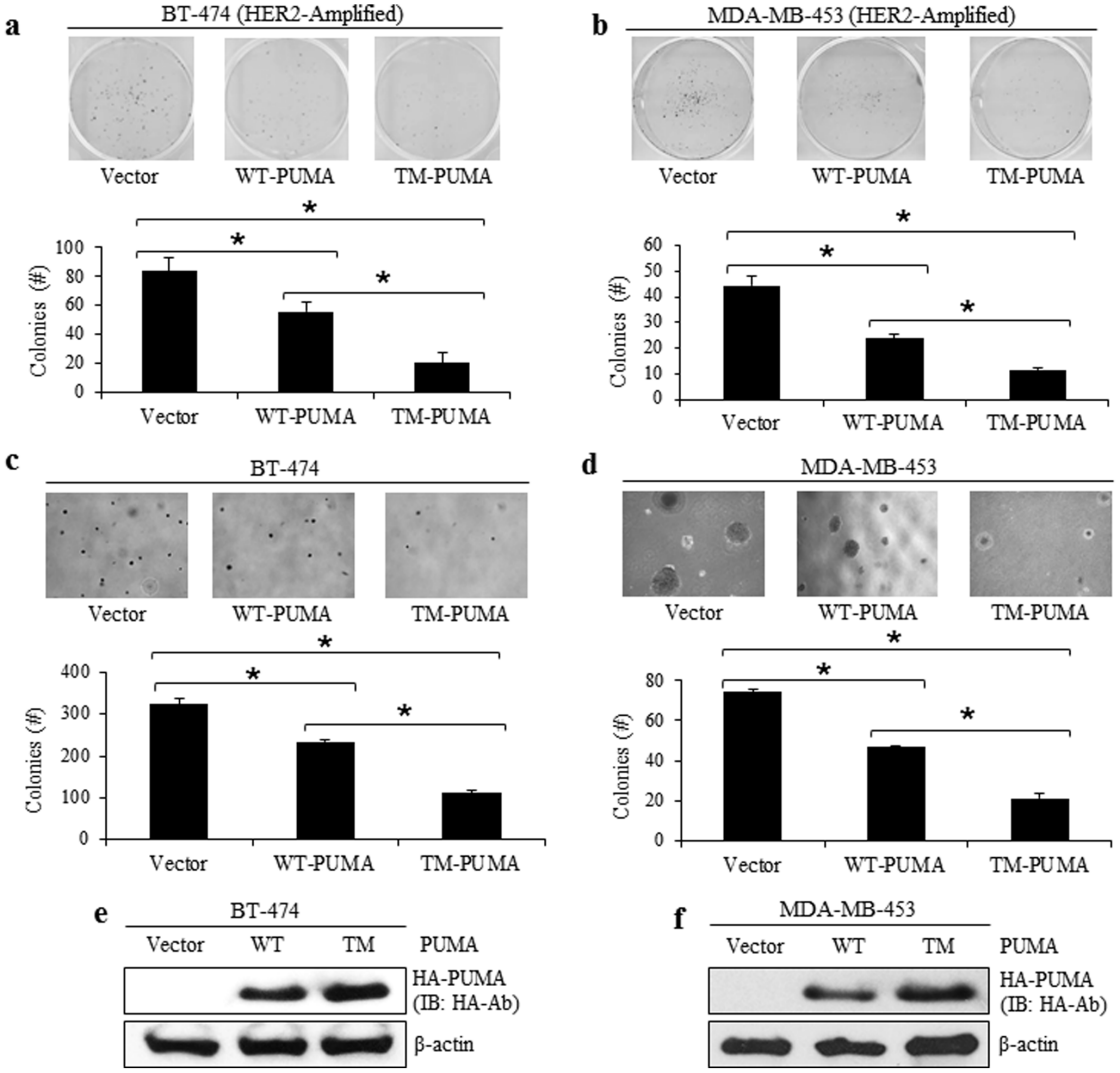
TM-PUMA Decreases Clonogenic Growth in HER2 Overexpressing Cells. BT-474 cells (a, c, e) and MDA-MB-453 cells (b, d, f) were transfected with an empty vector, WT-PUMA, or TM-PUMA for 48 hrs (e, f). Transfected cells were then seeded into 6-well plates either without (a and b) or with soft agarose (c and d) and incubated at 37°C for 14–21 days. Colonies were then counted, stained with crystal violet blue, and images were taken.

Figure 4 indicated that TM-PUMA had greater protein stability and greater protein levels in the mitochondria, which may indicate that TM-PUMA has an enhanced ability to promote apoptosis. To examine the effect of TM-PUMA on cell viability, we expressed an empty vector, WT-PUMA or TM-PUMA in two different HER2-overexpressing breast cancer cell lines, namely BT-474 (Figure 5e) and MDA-MB-453 (Figure 5f) cells, and monitored the ability of these cells to form colonies. As shown by the anchorage-dependent colony assay (Figures 5a and 5b), TM-PUMA significantly decreased colony formation compared to WT-PUMA, indicating that TM-PUMA had a stronger growth suppression than WT-PUMA. As expected, compared to the empty vector, WT-PUMA had a stronger propensity to decrease the colony formation ability of both cell lines. Both of these cells are aggressive and will grow independent of attachment. Therefore, a similar experiment was also performed with the same cell lines but using an anchorage-independent soft agarose colony assay. TM-PUMA significantly reduced soft agarose colony formation compared to WT-PUMA (Figures 5c and 5d). WT-PUMA also reduced colony formation compared to vector in both cell lines. Together, these data demonstrate that TM-PUMA has a greater effect than WT-PUMA on decreasing clonogenic growth of breast cancer cells and suggests tyrosine phosphorylation of PUMA decreases the ability of PUMA to suppress cell growth.

### TM-PUMA Induces Apoptosis to a Greater Degree than WT-PUMA

**Figure 6:**
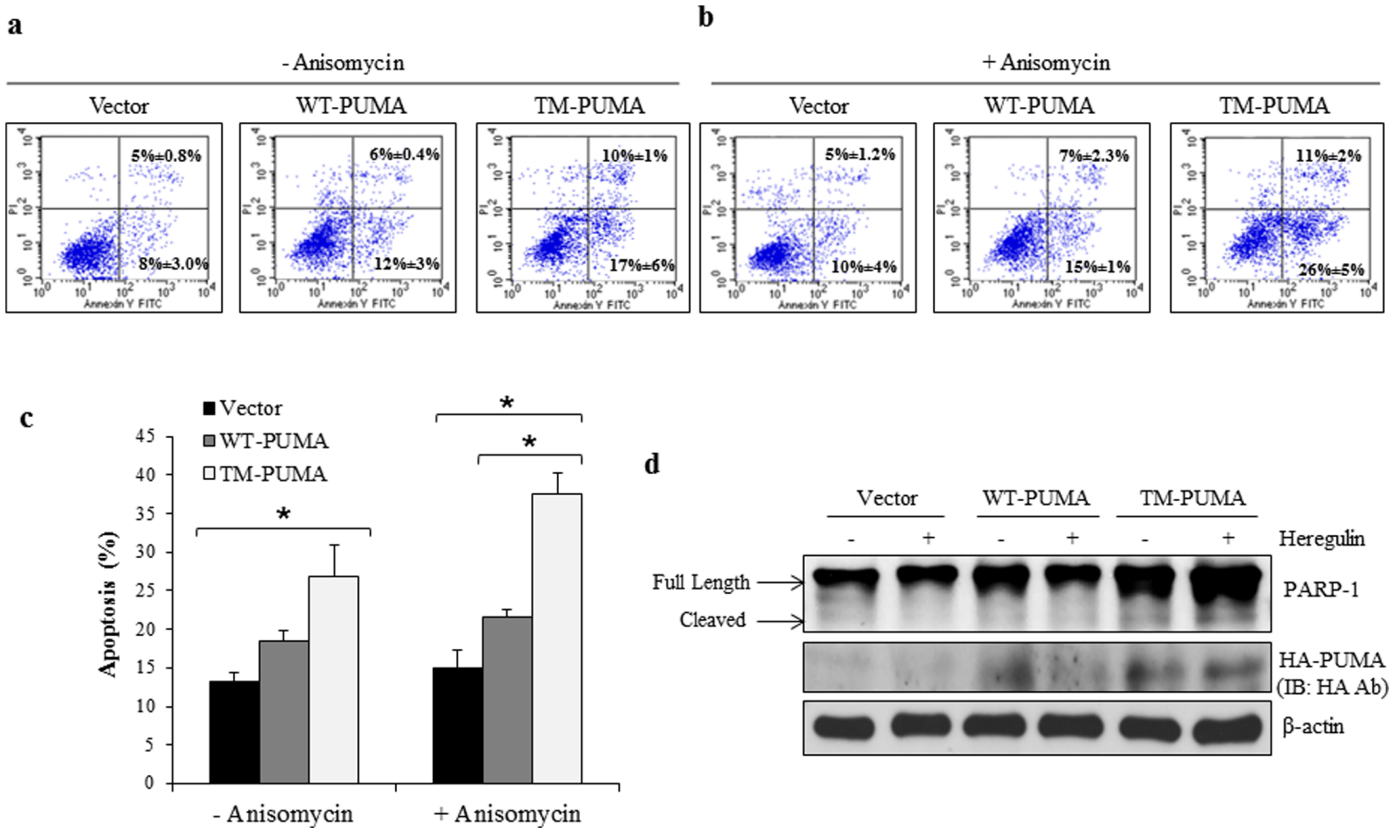
TM-PUMA Induces Apoptosis in HER2 Overexpressing Cells. a) and b) BT-474 cells were transfected with an empty vector, WT-PUMA, or TM-PUMA. Cells were treated with heregulin (100 ng/mL) and with or without anisomycin (25 ng/mL) for 16 hrs. Cells were detached and incubated with annexin-V-FITC and PI according to manufacturer’s instructions followed by analysis by flow cytometry. c) Graph representing measurements of apoptosis from (a and b). d) BT-474 cells were transfected with an empty vector, WT-PUMA, or TM-PUMA. Cells were incubated in serum-free medium for 16 hrs followed by treatment with heregulin (100 ng/mL) for 4 hrs. Cells were then lysed and total protein was subjected to immunoblotting with indicated antibodies.

We observed that TM-PUMA has a greater effect on cell growth than WT-PUMA in the context of HER2 overexpressing cells. However, whether this decrease in cell growth with TM-PUMA was due to enhanced apoptosis cannot be determined from analysis of the colony assays. To determine the effect of TM-PUMA on apoptosis, BT-474 cells were transfected with an empty vector, WT-PUMA, or TM-PUMA followed by treatment with heregulin to ensure HER2 activation. We then assessed the extent of apoptosis in the treated cells by the Annexin V binding assay using flow cytometry. Figures 6a and 6c show that TM-PUMA induced the greatest levels of apoptosis compared to WT-PUMA or empty vector. PUMA has been shown previously to sensitize cancer cells to treatment with apoptosis-inducing chemotherapeutic agents [21]. Therefore, we next assessed whether TM-PUMA could further enhance apoptosis in the presence of a low dose of anisomycin, an apoptosis inducer [35]. To this end, cancer cells were transfected with vector, WT-PUMA, or TM-PUMA followed by exposure to heregulin and anisomycin with subsequent assessment of Annexin V binding. As shown in Figures 6b and 6c, TM-PUMA expression significantly promoted apoptosis in the presence of anisomycin compared to vector and WT-PUMA. As expected, we observed modest increases in apoptosis in anisomycin-treated cells expressing vector or WT-PUMA compared to untreated cells.

To confirm the effects of WT-PUMA and TM-PUMA on apoptosis, cell lysates were analyzed by WB for the presence of PARP-1 cleavage. Consistent with the results of the Annexin V staining, the results revealed that TM-PUMA induced the greatest levels of cleaved PARP-1 (Figure 6d). Together, results presented in Figure 6 indicate TM-PUMA as a stronger apoptosis inducer than WT-PUMA and that tyrosine phosphorylation of PUMA reduces the ability of PUMA to promote apoptosis.

## Discussion

**Figure 7:**
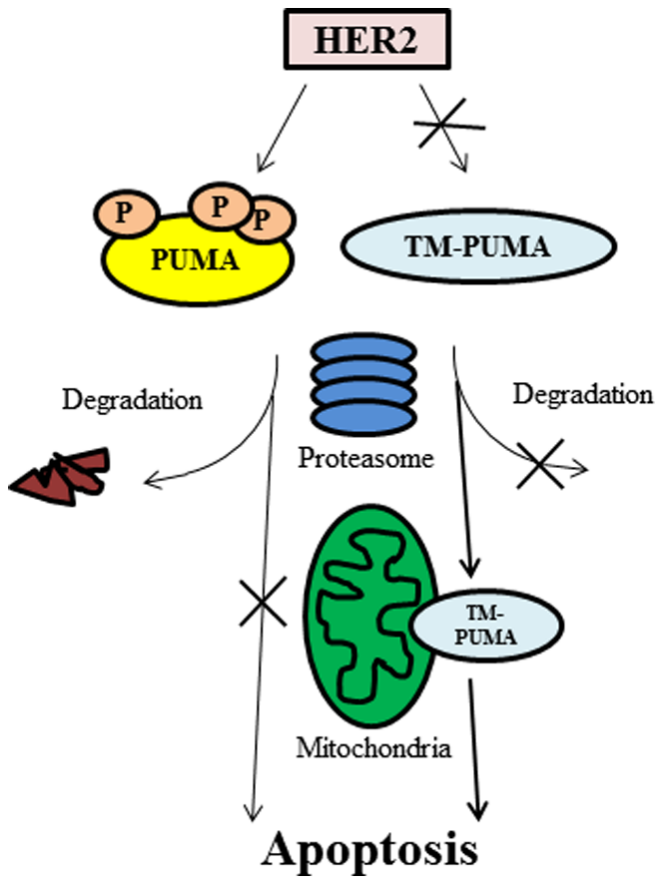
HER2 Downregulates PUMA by Phosphorylation. HER2 phosphorylates PUMA on three tyrosine residues leading to degradation by the proteasome reducing apoptosis. TM-PUMA, which cannot be phosphorylated by HER2, has an extended half-life and localizes to the mitochondria and promotes apoptosis.

We report in this study that HER2 directly phosphorylates PUMA and this leads to PUMA degradation and suppression of apoptosis (Figure 7). This finding is novel and significant because HER2-mediated negative regulation of PUMA is direct and distinctly different than previously reported mechanisms by which HER2 can indirectly antagonize apoptosis. Furthermore, PUMA was recently shown to be required for HER2 inactivation-induced apoptosis [36] and our data suggest a direct method whereby HER2 downregulates PUMA protein levels. Overexpression of HER2 occurs in 15–20% of breast cancers and is a marker for poor patient outcome [2]–[4]. HER2 promotes several characteristics common to cancer cells including activation of downstream signaling, especially PI3K-AKT and MAPK pathways, promotion of cell division, inhibition of apoptosis, and promotion cell motility among others [4]. HER2 regulation of apoptosis can be mediated by indirect upregulation of anti-apoptotic proteins such as Bcl-2 and Bcl-xL [7], [11]. Also, HER2-mediated activation of AKT leads to phosphorylation and down regulation of Bad, another pro-apoptotic BH3-only protein [10]. In addition, our lab recently found that EGFR, another ErbB family member, could physically interact with PUMA, which prevented PUMA localization to the mitochondria and suppressed apoptosis [26]. These studies and the current data indicate that ErbB receptor tyrosine kinases regulate apoptosis at multiple levels including indirect regulation, via downstream signaling components, and direct regulation, such as post-translational regulation of PUMA by HER2.

The current study is the first evidence that PUMA can be phosphorylated on tyrosine residues. In 2010, Fricker et al. first observed that PUMA protein could incorporate labeled ^32^P suggesting it could be phosphorylated [31]. Their analysis indicated it was primarily serine residues that were phosphorylated with serine-10 being the major target. Mutation of serine-10 to alanine resulted in a PUMA mutant protein that showed greater induction of apoptosis than wild type PUMA, which was the result of an extended protein half-life [31]. Investigators were not able to identify any kinase that mediated serine-10 phosphorylation of PUMA. However, another group soon found that IL-3 signaling induced phosphorylation at serine-10 and enhanced PUMA protein degradation [34]. These results ultimately identified IκB kinase-1 (IKK1) as the kinase that directly phosphorylates serine-10 of PUMA in response to IL-3 signaling [34]. Results of the current study and these recent studies indicate phosphorylation and degradation of PUMA provide cells an escape from apoptosis under conditions replete with growth-promoting signals. Future identification of other kinases that phosphorylate PUMA will provide a greater understanding of what cellular contexts PUMA phosphorylation may be important.

PUMA expression enhances apoptosis induction by chemotherapy [21]. Chemotherapy treatment has been shown to induce PUMA expression in breast cancer cells [21], [37], [38] and PUMA is a primary mediator of apoptosis in response to tamoxifen in breast cancer cells [39]. Forced HER2 expression in HER2-negative cells suppressed apoptosis and induced tamoxifen resistance because of increased expression of anti-apoptotic proteins such as Bcl-2 [7]. Here we show that HER2 can additionally regulate apoptosis by phosphorylating tyrosine residues on PUMA leading to its degradation. HER2-mediated down regulation of PUMA via phosphorylation and HER2-mediated upregulation of anti-apoptotic Bcl-2 proteins creates a cellular environment highly resistant to apoptosis.

We also observed that TM-PUMA, which is resistant to HER2 phosphorylation, has increased protein stability and enhanced induction of apoptosis by pro-apoptotic agents in the presence of HER2 overexpression compared to WT-PUMA. Evidence for the benefits of targeting Bcl-2 family proteins is accumulating as BH3-only mimicking agents can promote apoptosis and enhance apoptosis with chemotherapy [40]–[42]. Specifically, ABT-737 was recently observed to sensitize primary breast tumors overexpressing Bcl-2 to chemotherapy [42]. Also, tumor-specific PUMA gene transfer enhanced radiosensitivity of breast cancer cells, although this result was in cells that do not overexpress HER2 [43]. TM-PUMA would likely be a more beneficial gene therapy in HER2-overexpressing cells as our results suggest HER2 overexpression decreases WT-PUMA-induced apoptosis. Together, these results indicate that targeting Bcl-2 family proteins in addition to chemotherapy may provide greater breast cancer cell death.

Our data, and others [31], [34], indicate that inhibition of the proteasome extends the half-life of PUMA (Figure 4) whereas inhibition of lysosomal proteases has little effect on PUMA degradation [33]. This would suggest the proteasome is the primary mediator of PUMA protein degradation. Considering that our TM-PUMA, which cannot undergo tyrosine phosphorylation, is not degraded by the proteasome it is likely that tyrosine phosphorylation allows for further modifications that target PUMA to the proteasome. Given the nascent understanding of PUMA post-translational modifications, further investigation is needed to elucidate the specific modifications required to target PUMA to the proteasome and to determine what enzymes make these modifications.

To extend the findings reported in this study, there should be further investigation into whether other tyrosine kinases can phosphorylate PUMA as this could indicate other cellular and disease contexts in which PUMA phosphorylation is important. Also, the role of phosphorylation of each tyrosine residue on PUMA should be determined. Our results indicate all three tyrosine residues can be phosphorylated by HER2 but the role of each tyrosine residue on PUMA function cannot be determined herein. Lastly, it should be determined whether tyrosine phosphorylation-resistant TM-PUMA and BH3 mimetics can enhance the effectiveness of chemotherapeutics in HER2-overexressing breast tumors. These future studies will elucidate the importance of BH3-only proteins, especially PUMA, in breast cancer and advance the search for therapeutic targets in HER2-positive tumors.
